# Cutaneous Squamous Cell Carcinoma in the Age of Immunotherapy

**DOI:** 10.3390/cancers13051148

**Published:** 2021-03-08

**Authors:** Yosuke Ishitsuka, Yuma Hanaoka, Atsushi Tanemura, Manabu Fujimoto

**Affiliations:** Department of Dermatology Integrated Medicine, Osaka University Graduate School of Medicine, 2-2 Yamadaoka, Suita, Osaka 565-0871, Japan; 127hanaoka@derma.med.osaka-u.ac.jp (Y.H.); tanemura@derma.med.osaka-u.ac.jp (A.T.); fujimoto@derma.med.osaka-u.ac.jp (M.F.)

**Keywords:** atopic dermatitis, cancer immunoediting, cornification, genetic diseases, immune checkpoint inhibitor, immunodeficiency, Langerhans cells, malignant melanoma, MAPK signaling, PD-1, SCC, TGF-β

## Abstract

**Simple Summary:**

Cutaneous squamous cell carcinoma (cSCC) is the second most prevalent skin cancer globally. Immunosuppression raises cSCC incidence rates, while high immunogenicity of the cutaneous tissue enables topical immunotherapy. Intriguingly, expanded applications of programmed death-1 (PD-1) blockade therapies have revealed cSCC to be one of the most amenable targets. These clinical observations prompted us to redefine cSCC biology and review current knowledge about cSCC from multiple viewpoints that involve epidemiology, clinicopathology, molecular genetics, molecular immunology, and developmental biology. This synthesis reinforces the following hypothesis: PD-1 blockade effectively restores the immunity specially allowed to exist within the fully cornified squamous epithelium, that is, the epidermis.

**Abstract:**

Cutaneous squamous cell carcinoma (cSCC) is the second most prevalent skin cancer globally. Because most cSCC cases are manageable by local excision/radiotherapy and hardly become life-threatening, they are often excluded from cancer registries in most countries. Compared with cutaneous melanoma that originates from the melanin-producing, neural crest-derived epidermal resident, keratinocyte (KC)-derived cancers are influenced by the immune system with regards to their pathogenetic behaviour. Congenital or acquired immunosurveillance impairments compromise tumoricidal activity and raises cSCC incidence rates. Intriguingly, expanded applications of programmed death-1 (PD-1) blockade therapies have revealed cSCC to be one of the most amenable targets, particularly when compared with the mucosal counterparts arisen in the esophagus or the cervix. The clinical observation reminds us that cutaneous tissue has a peculiarly high immunogenicity that can evoke tumoricidal recall responses topically. Here we attempt to redefine cSCC biology and review current knowledge about cSCC from multiple viewpoints that involve epidemiology, clinicopathology, molecular genetics, molecular immunology, and developmental biology. This synthesis not only underscores the primal importance of the immune system, rather than just a mere accumulation of ultraviolet-induced mutations but also reinforces the following hypothesis: PD-1 blockade effectively restores the immunity specially allowed to exist within the fully cornified squamous epithelium, that is, the epidermis.

## 1. Introduction and Overview

### Cutaneous Squamous Cell Carcinoma (cSCC) in the Age of Immunotherapy

Cutaneous neoplasms, benign or malign, are defined as aberrantly accumulated patches of mutated or altered cells [1,2]. Key genomic features of malignancy are readily identified in cSCC precursor actinic keratosis (AK) [3], benign squamous neoplasms [4] or even normal aged skin [5]. Clinical observations suggest that host immune responses serve as selective pressure and ultimately shape the outcome of malignant progression [6]. A prime clinical example is demonstrated in organ transplant recipients [7]. Both long-term immunosuppression and the graft-versus-host reaction (GVHR) [7,8]/lichenoid tissue reaction [9,10], which attacks the germinal layer of the epidermis, fuel the development of SCCs. Keratoacanthomas (KAs), which are self-involuting squamous cell epitheliomas, develop in individuals with transforming growth factor-beta (TGF-β) signaling haploinsufficiency [11]. Topical immunotherapy for cSCC precursors is about to become a mechanism-based regimen [12]. These clinical observations clearly delineate the profound impact of the immune system on the cSCC pathology.

Although most cSCC cases are generally nonlethal and manageable with surgical excision or radiotherapy [13], no systemic therapies have been approved for unresectable, life-threatening advanced diseases. Before the emergence of receptor tyrosine kinase inhibitors (RTKIs), classical, platinum-based treatment regimens were a common practice [14]. An epidermal growth factor receptor (EGFR) blockade offers a legitimate, biology-based option but does not warrant durable responses [14]. Cancer immunotherapy had only been implemented in the past decade, beginning with the introduction of immune checkpoint inhibitors (ICIs) into the clinical practice. In the realm of dermatology, ICIs achieve durable responses in advanced cutaneous malignant melanomas, turning the deadly disease into a manageable ailment [15]. Dermatologists have also experienced the consequences of aberrantly activated immune responses, i.e., immune-related adverse events (irAEs) [16], whose cutaneous involvement is frequent [17]. Only recently, the extended application of ICIs has finally made us realize that cSCC could be an amenable target [18,19], providing another reliable treatment option for advanced, potentially lethal cSCCs [18,20]. Because ICIs reactivate immune responses in the peripheral tissue, we try to address outstanding questions, “what makes cSCC an amenable target?,” or “why the skin is highly immunogenic?” This manuscript reviews current knowledge about cSCC from multiple points of view and discuss rational management strategies for this common neoplasm in the age of immunotherapy.

## 2. Epidemiology of cSCC

Basal cell carcinoma (BCC) and cSCC, which are often referred to as keratinocyte (KC) cancer in aggregation [21], are the most common skin cancers globally, and cSCC is the second most common skin cancer [22]. Because most cSCC cases tend to be metachronous but nonlethal [13,23], this entity is often excluded from cancer registries, and most statistical data are based on surveys or treatment data from subsets of national populations [24]. cSCC commonly arises from its precursor actinic keratosis in sun-damaged skin. The highest incidence is observed in fair-skinned people who have fair eye, skin and hair color, as well as people with inborn errors in melanin synthesis, i.e., oculocutaneous albinism [25]. Based on the cSCC incidence in dark-skinned people (African and Asian heritage), high ambient ultraviolet (UV) radiation (UVR) levels confer greater risk [24]. An Australian systematic review suggested an estimated cSCC incidence of as high as 2% in 2002, with the highest rates recorded in Queensland [26]. A recent registry-based data in the Netherlands revealed incidence rates of 107.6 per 100,000 people–year (PY) for men and 68.7 per 100,000 PY for women, which correspond to US-standardized rates [27]. Given that increasing population ageing is expected to further increase the incidence of cSCC, there is a need for mechanism-based, legitimate management strategies that can accommodate the elderly [22].

## 3. Clinicopathological Stratification of cSCC

### 3.1. Clinical and Histopathologic Stratification

The low differentiation capacity of epithelial cells corresponds to the loss of cell polarity and cell–cell adhesion and the gain of invasive and metastatic potential, often accompanied by epithelial–mesenchymal transition (EMT) [28]. At the molecular level, poorly differentiated cSCC (desmoplastic/spindle cell/sarcomatoid variant) acquires expression of the mesenchymal intermediate filament vimentin [29,30] and typically loses the adhesion molecule E-cadherin [29,31,32]. Skin cancer tissue comprises tumor cells and stromal responses, and malignant biological behaviors of skin neoplasms can be differentiated on the basis of appearance [33]. The best example is likely KA, which is also known as molluscum sebaceum [34], or Sabouraud’s ‘button epithelioma’ [35]. KA is a well-differentiated cSCC subtype that exhibits a symmetric, crateriform appearance and a large central keratin plug with pronounced, well-differentiated squamous cell proliferation, and it often displays spontaneous regression [34,35]. Well-differentiated verrucous carcinoma harbors low metastatic potential, whereas highly infiltrative desmoplastic cSCC possesses higher risks of recurrence and metastasis [13].

#### 3.1.1. Factors Associated with Local Recurrence and Metastasis

In general, cSCC carries an excellent prognosis, but a subset of tumors has a high risk of poor outcomes, including metastasis and mortality rates of 3.7 and 2.8%, respectively [36]. Because 70% of deaths are attributable to unresectable locoregional disease rather than distant organ metastases [37], clinicopathological risk stratification and the early detection of lymph node metastases are mandatory [13].

Tumor diameter >2.0 cm is the risk factor most highly associated with disease-specific death, as it confers a 19-fold higher risk of death from cSCC than tumor diameter <2 cm [13]. Tumor depth is also associated with local recurrence and metastasis. Specifically, Breslow thickness >2 mm and invasion into deep tissues (fascia, muscle, perichondrium or periosteum) are known factors [13]. Although the perineural involvement of cSCC is uncommon, large-caliber nerve (>0.1 mm) involvement is associated with nodal metastasis and mortality [13]. Recurrent cSCCs carry a much worse prognosis and a higher risk of spread to regional lymph nodes and distant metastases, as indicated by rates of 45% for ear cSCC and 32% for lip cSCC [13]. cSCCs that arose on particular anatomic sites, such as the ears and lips, have been reported to have local recurrence rates exceeding 10% in the absence of non-Mohs modalities [13]. Scar tissue caused by chronic inflammation, such as leg ulcers, burn scar, radiation dermatitis and discoid lupus, can reportedly elevate the rate of metastasis to 26% [13]. Immunosuppression compromises immunosurveillance and increase the risks of local recurrence and metastasis [13].

#### 3.1.2. Staging Systems for cSCC

Currently, cSCC staging systems from the American Joint Committee on Cancer (AJCC) [38]/Union for International Cancer Control (UICC) [39] staging manual and Brigham and Women’s Hospital (BWH) [40] are available. Because cSCCs have been excluded from the Surveillance, Epidemiology, and End Results tracking program and cancer registries, the AJCC/UICC classification does not have access to such population-based data [36,40]. Moreover, the eighth edition of the AJCC staging system, which entered clinical use in January 2018, includes an updated classification on only head and neck cases [38]. From a practical point of view, stratifying the high-risk group that require further checkup is essential [40]. To address this issue, the BWH system was proposed as an alternative tumuor staging system [40]. Studies on risk stratification performance between the BWH and AJCC systems revealed that the former offers superior distinctiveness, homogeneity and monotonicity [41]. Therefore, the BWH system could avoid the inappropriate upstaging of low-risk cases [36]. Overall, the BWH system appears to be a practical risk-stratification system that offers a legitimate follow-up strategy at the initial time of diagnosis (Table 1).

## 4. Etiology of cSCC: Exogenous Factors

As mentioned previously, UVR (both UVB and UVA) is a bona fide major risk factor for cSCC [22,42]. The use of a tanning bed increases the risk of KC cancer in a UV dose-dependent manner. Incremented or occupational sun exposure is more directly related to the incidence of cSCC than to that of BCC [22]. Immunosuppression is another important risk factor, and long-term immunosuppressive therapy in solid-organ transplant recipients increases the risk of cSCC by a factor of 100 compared with that in the general population [42]. Infection by oncogenic human papillomavirus (HPV), which is believed to inhibit DNA repair, is also associated with cSCC [24,43]. Human immunodeficiency virus (HIV) infection decreases peripheral blood CD4 counts and elevates the incidence of KC cancer [44]. Exposure to polyaromatic hydrocarbons or arsenic represents a classic environmental/occupational risk factor [24].

## 5. Etiology of cSCC: Endogenous Factors

In addition to UVR sensitivities determined by skin types, genetic factors predispose individuals to cSCC. Identified links between genotype and phenotype allow us to understand the pathomechanism of cSCC development. In principle, inborn errors in DNA repairing or immune signaling pathways are predisposing factors for cSCC (Table 2).

### 5.1. Defective DNA Repair

Defective DNA repair impairs genome maintenance and increase the mutational load (Table 2) [45,46]. The classic example is xeroderma pigmentosum, which is caused by the failure of nucleotide excision repair following UV damage [47]. Deficiencies in DNA interstrand cross-link repair cause the bone marrow (BM) failure syndrome Fanconi anemia (FA) [48]. Compromised telomerase function leads to dyskeratosis congenita, which is accompanied by dysplastic nails and oral leukoplakia, and typical progeria features [45,49]. Germline DNA mismatch repair mutations cause Muir–Torre syndrome, which is associated with KAs and sebaceous neoplasia/internal malignancies [50].

### 5.2. Primary Immunodeficiency

The immune system is the site of various genotoxic stresses that occur during immune system maturation and immune responses [51]; DNA-altering mechanisms are important in the development of T and B cells, as observed in V(D)J recombination, immunoglobulin class switch recombination and the generation of somatic hypermutations [52,53]. Therefore, it is not surprising that primary immunodeficiencies lead to autoimmunity and increased susceptibility to infections or malignancies [51]. Regarding the susceptibility to cSCC, impaired surveillance against oncogenic viruses or mutated KC is considered responsible [51,54].

#### 5.2.1. Epidermodysplasia Verruciformis (EV)

EV is characterized by increased susceptibility to cutaneous beta HPV infection and cSCC [55] in association with the global suppression of adaptive cell-mediated immune responses in the skin [56,57,58,59,60]. Loss-of-function (LOF) mutations in evolutionary conserved transmembrane protein channel-like gene family members *EVER1/EVER2* represent the classic predisposing factor [61] that accounts for 75% of cases [55]. In addition to genetic factors [55], patients with severe combined immune deficiency (SCID) [62] or HIV infection [63] can also display EV-like phenotypes. Moreover, EV develops in patients who have undergone BM transplantation, suggesting that non-BM-dependent, innate immune components could be the disease driver. This raises the possibility that an impaired KC-intrinsic innate immune response is responsible for the phenotype [62], as suggested previously [56,57,64]. Accordingly, it was recently uncovered that LOF mutations in calcium and integrin binding 1, which forms a complex with EVER1/EVER2 and inhibits intracellular HPV expansion in KCs, underlie EV phenotypes [65].

#### 5.2.2. GATA Binding Protein 2 (GATA2) Deficiency/Monocytopenia and Mycobacterial infection (MonoMAC) Syndrome

The interleukin-12 (IL-12)/IL-23p40/interferon-gamma axis control adaptive cell-mediated immune responses. Defective functioning of monocyte/B cell/natural killer cell in GATA2 deficiency [66,67] is considered responsible for sporadic MonoMAC syndrome [68].

#### 5.2.3. WHIM Syndrome

Germline gain-of-function (GOF) mutations in C-X-C chemokine receptor type 4 cause warts, hypogammaglobulinemia, infections and myelocathexis (WHIM) syndrome [69]. C-X-C chemokine ligand 8 (CXCL8)/CXCR2-mediated and CXCL12/CXCR4-mediated signaling controls BM release of polymorphonuclear neutrophils (PMNs). GOF mutations in CXCR4 are considered to inhibit the PMN release from BM and compromise primary immune responses [70].

#### 5.2.4. Hyper-IgE Recurrent Infection Syndrome (HIES)

The pathologic characteristics of atopic dermatitis (AD) involve elevated serum IgE levels and impaired cell-mediated immune responses [71], and HIES phenotypes somewhat resemble AD. As recurrent staphylococcal infections in AD implies, in most cases defects in T helper 17 (Th17) cell differentiation [72] underlie HIES pathology. IL-6 signaling [73], its downstream target signal transducer and activator of transcription 3 (*STAT3*) [74] and retinoic acid receptor-orphan receptor-γt [75,76] promote Th17 cell differentiation. LOF mutations in *STAT3* [77], IL-6 receptor [78] and IL-6 signal transducer [79] result in HIES.

### 5.3. Impaired TGF-β Signalling Pathway

The TGF-β signaling pathway is a fundamental biological pathway that regulates several cellular processes in the skin, including epidermal differentiation [80,81] and carcinogenesis [31,82,83]. TGF-β signaling inhibits DNA synthesis [84], mediates DNA damage responses [85] and suppresses genomic instability [86,87]. The hypomorphic allele of the type I TGF-β receptor TGFBR1*6A is known as a low-penetrance allele that predisposes individuals to breast cancer, ovarian cancer, hematologic malignancies [88] and colorectal cancer [89]. Likewise, LOF mutations in *TGFBR1* cause self-healing cSCC-like lesions that resemble KAs in Ferguson–Smith disease (FSD) [90], similarly as the pan-TGF-β–blocking antibody fresolimumab (GC1008) [91,92]. Germline [93]/somatic [94,95] LOF *TGFBR2* mutations are associated with tumorigenesis, and advanced cSCCs exhibit low TGFBRII expression levels [31,96]. In principle, TGF-β confers cancer resistance.

#### FSD in the Context of Cancer Immunoediting

Self-healing multiple squamous epitheliomas in patients with FSD [11], or alternatively TGF-β blockade [91,92], could give us interesting insights. Adaptive immunity restrains cancer cells in a state of dormancy [6]. However, some clones overcome the selective pressure, and resistant clones evolve. This theoretical framework represents cancer immunoediting [6,97]. We try to illustrate FSD in the context of cancer immunoediting.

Germline haploinsufficiency in *TGFBR1* (FSD) likely affects cutaneous cancer immunosurveillance. Epidermal Langerhans cells (LCs) present tissue antigens to draining lymph nodes in steady state [98], and the TGF-β signaling pathway maintains epidermal LC networks in mice [99]. Therefore, the first possibility is that patients with FSD exhibit impaired epithelial antigen priming.

**Figure 1:**
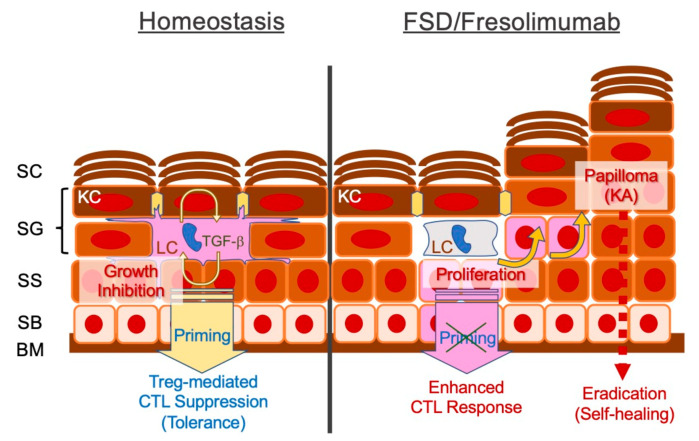
Schematic representation of defective transforming growth factor-beta (TGF-β) signaling in the epidermis and possible pathomechanisms of self-healing squamous epithelioma observed in Ferguson–Smith disease (FSD) or after treatment with the TGF-β antibody fresolimumab. Epidermal Langerhans cells (LCs) require an autocrine/paracrine loop of TGF-β. LCs establish an intercellular network of differentiated epidermal layers, induce tolerance in steady state and protect against potentially harmful cytotoxic T cell (CTL)-mediated immune responses. Disrupted TGF-β signaling may augment keratinocyte DNA synthesis, facilitating the formation of epitheliomas (papillomas), whereas unleashed CTLs eradicate the tumor. BM, basement membrane; SB, stratum basale; SS, stratum spinosum; SG, stratum granulosum; SC, stratum corneum.

TGF-β signaling primarily suppresses cytotoxic T lymphocytes (CTLs)-mediated tumoricidal activity [100]. Conversely, tumor cell-derived TGF-β augments metastatic potential of cSCC [31,82,101], possibly producing an immnosuppressive microenvironment [31]. Therefore, the TGF-β1–rich microenvironment primarily leads to the CD4^+^ regulatory T cell (Treg)-mediated suppression of tumoricidal activities [100]. Possible mechanisms in FSD are as follows: (i) impaired epidermal LC network maintenance compromises tumor surveillance [102], promotes KC proliferation [80,81] and gives rise to squamous epitheliomas [103]; (ii) impaired Treg effector function augments tumoricidal activity [11]; and (iii) impairment of the autocrine or paracrine loop of TGF-β signaling inhibits EMT or immunoevasion, respectively [31,82,96,101] (Figure 1).

The spontaneous regression of cancers, which is likely mediated through natural resistance [104], is a common phenomenon that has been described for almost a century [104,105,106,107]. However, applying the cancer immunoediting concept [6,97] to TGF-β signaling may provide fascinating insights for clinical cancer research.

## 6. Beyond Targeted Therapy

### 6.1. Any Druggable Targets in cSCCs?

Until recently, there had been approved systematic therapy for patients with cSCC. Although EGFR inhibition remains a legitimate, biology-based option with substantial treatment efficacy, a relatively short progression-free survival (<8 months) in a phase 2 trial suggests drug resistance [14].

The personalized medicine concept has been developed in recent decades, and the advent of massive parallel sequencing combined with single-cell technology has accelerated the comprehensive understanding of tumor microenvironments from multiple biological aspects [108,109]. Moreover, multi-omic analyses started to uncover the trajectory of clonal events during cancer evolution [110]. These data-driven biomedical methods may pinpoint a yet-to-be-defined Achilles’ heel of cSCC, in line with the effects of trastuzumab (human epidermal growth factor receptor 2 inhibitor) in breast cancer [111], cetuximab (EGFR inhibitor) in colorectal cancer [112], imatinib (RTKI) in dermatofibrosarcoma protuberans [113] and dabrafenib (BRAF inhibitor)/trametinib (MEK inhibitor) in malignant melanoma [114]. However, investigations to date have revealed that recurrently altered genes [3,115,116,117] (Table 3), as well as the clonal selection, are conserved features of human malignancies. In this chapter, we would like to review the history of melanoma research that has yielded successful therapeutic measures targeting against the mitogen-activated protein kinase (MAPK) signalling cascade [15,114,118,119]. This comparison may facilitate an in-depth understanding of cSCC in the age of immunotherapy.

### 6.2. Genetic Component of Malignant Melanoma

The melanocyte, uniquely located in the basal epidermal layer, constitutes an important part of protection against UVR by supplying neighboring basal KCs with melanosomes, which are melanin-laden organelles [120]. Melanoma, a neoplasm of transformed melanocytes, has been the subject of intensive research because of its high lethality [121,122]. Inborn errors in genes that control the G1 checkpoint, such as cyclin-dependent kinase inhibitor 2A (*CDKN2A*), enhance cellular proliferation and result in familial melanomas [123,124,125]. Because high proliferation rates represent a hallmark feature of cancers, this type of germline variant also gives rise to higher incidence rates of non-melanoma malignancies, such as pancreatic cancer (Table 4) [123].

#### 6.2.1. MAPK Signaling Cascade and RASopathy

The MAPK signaling cascade regulates a wide range of cellular responses, including cell cycle regulation [126]. Various external stimuli, particularly ligand binding with membrane-bound growth factor receptors, activate MAPK signaling [126]. The *RAS* oncogene, a GTPase [127], is the first messenger of this intracellular signaling cascade [128]. Upon binding with GTP, RAS then recruits the RAF kinase to the plasma membrane [129], triggering a series of downstream intracellular phosphorylation events [126,127]. It is noteworthy that compared with cancer-prone LOF G1 checkpoint mutations [123,125,130,131], the MAPK signaling cascade is indispensable for normal embryonic development in mice [132,133]. This is in accordance with multi-system developmental anomalies termed RASopathies, which are caused by germline MAPK-activating mutations in humans (Table 5) [134].

#### 6.2.2. Germline BRAF Mutations and Cardio-Facio-Cutaneous (CFC) Syndrome

CFC syndrome is a RASopathy associated with germline mutations in *KRAS* [135], *BRAF* (non-V600E) [135,136] and *MAP2K1* (*MEK1*)/*MAP2K2* (*MEK*2) [136]. Despite the presence of multiple melanocytic nevi (MNs) [134,137], CFC-associated *BRAF* variants [135,136] do not increase the incidence of melanoma or cSCCs (Table 5). Moreover, CFC-associated germline *BRAF* variants do not necessarily lead to MAPK activation [136], meaning that the clinical phenotypes do not necessarily reflect the degrees of MAPK signaling cascade activation [136]. This is in stark contrast with cancer-associated constitutively active somatic *BRAF*^V600E^ mutations [138]. Thus, it is worthwhile to clarify why *BRAF*^V600E^ is the selfish gene [139] that drives the clonal evolution of transformed melanocytes [140].

#### 6.2.3. Evolutionary Trajectory of Melanocytic Neoplasms

The MN, commonly known as the pigmented mole, is a benign melanocytic neoplasm. Because MNs present heterogeneous histopathologic features, the premise that all MNs are pre-malignant has been a subject of debate among dermatologists and dermatopathologists [141,142,143,144]. In particular, the term “dysplastic nevus (DN),” which was initially given for MNs in patients with familial melanoma [143], has been the subject of debate and the source of confusion for clinical practitioners [141,142]. This dispute finally led to the National Institutes of Health recommendation that DN should no longer be used for histopathological diagnosis in 1992 [145]. However, a molecular genetics study provided important evidence that could reconcile the controversy. Specifically, MNs harbor *BRAF*^V600E^ mutations with a similar frequency as melanomas [146], suggesting that the accumulation of activating mutation is the early neoplastic event of MN development [147]. Evidence from animal studies further support this notion. For instance, a study using a zebrafish model revealed that the *BRAF*^V600E^ mutation is sufficient to promote MN formation [148]. It was also demonstrated that the early clonal events (acquisition of *BRAF*^V600E^), with concomitant loss of tumor suppressors such as tumor protein 53 (*TP53*) [148,149] or phosphatase and tensin homolog (*PTEN*) [150], drive clonal evolution in cooperation with UVR [149]. Recent human studies essentially confirmed these findings. Unequivocally benign MN lesions exclusively harbored the *BRAF*^V600E^ mutation, whereas the majority of MNs categorized as intermediate were enriched with mutations in *NRAS*, *CDKN2A* or telomerase reverse transcriptase [151]. Despite the clonal selection at the earlier stage of progression, *PTEN*/*TP53* mutations were found only in advanced primary melanomas, and copy-number alterations became prevalent in invasive melanomas [151], all of which are the universal features of malignant progression. The comparison between the RASopathies and the trajectory analysis of melanoma reminded us of the antagonistic pleiotropy or the cancer field [104] theories. In short, the tissue homeostasis is the product of well-designed gene expression program. The reverse is also true; the cancer tissue (or developmental disorders) can result either from aberrations in designing (gene sequences) or execution (gene expression).

### 6.3. Genetic Mosaicism and the Gene Expression Programme

Chromatin regulators are frequently mutated in cancers. These mutations could modify chromatin and thus reprogram gene expression [152]. These adaptive, plastic but heritable cellular responses are indispensable for the development of organs [153], senescence [154] and immune responses [155], as well as cancers [156]. In this light, it was experimentally demonstrated that the neural crest progenitor transcription factor sex-determining region Y-box 10 converts *BRAF*^V600E^-expressing melanocytes (MNs) into melanomas [157]. Melanoma with *BRAF*^V600E/K^ (or other mutations) are sensitive to the targeted therapy because of these specific activating mutations that are not typically found in cSCC [158]; while they can be found in colon cancer [159], it is true that the activation of other pathways such as EGFR are responsible for lack of activity for single agent BRAF targeted therapies.

**Figure 2:**
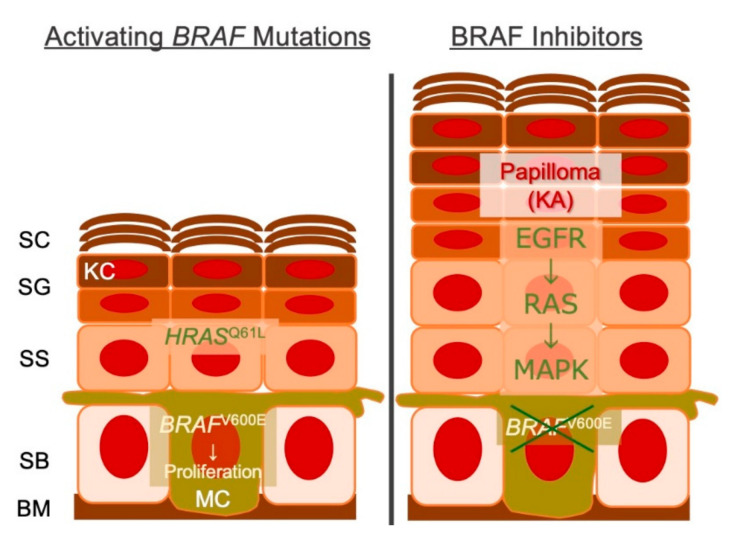
Schematic representation cSCC/KAs secondary to BRAF inhibition. Melanocytes with *BRAF*^V600E/K^ (or other mutations) are sensitive to the targeted therapy, whereas KC with *HRAS*^Q61L^ (or other mutations) are not. BM, basement membrane; SB, stratum basale; SS, stratum spinosum; SG, stratum granulosum; SC, stratum corneum; KC, keratinocyte; MC, melanocyte; KA, keratoacanthoma.

A previous report showed that the BRAF inhibitor vemurafenib provokes the development of cSCCs/KAs because of the paradoxical activation of MAPK located distally [158]. Mutations in *RAS* oncogene promote cellular transformation fueled by the acquisition of cell cycle-altering passenger mutations [158]. Because up to 60% of the squamous epitheliomas are considered to harbor MAPK-activating *HRAS*^Q61L^ mutations [158], pr-eexisting KC mutations could determine the outcome (Figure 2). Mechanistically, when EGFR-RAS signaling is activated, inhibitor binding induces conformational changes in the RAF kinase domain, which in turn causes the wild-type RAF isoform to dimerize, translocalize to the membrane, and interact with RAS-GTP [160]. Although these observations clearly illustrate the dependence on specific oncogenes in specific epidermal cell-lineages, caution should be exercised to avoid the oversimplification. Let us take developmental disorders caused by the genetic mosaicism of oncogenes, as with germline mutations in the RASopathy, somatic mutations in the developmental pathway can render very different consequences. Postzygotic HRAS/KRAS mutations produce the organoid nevus (benign hamartoma) called nevus sebaceous, and more severe consequences can manifest as the developmental anomaly termed Schimmelpenning syndrome, in which aberrations in the ectodermal development are thought to cause cerebral, ocular and skeletal defects [161]. By analogy, somatic mutations in oncogenic fibroblast growth factor receptor 3 (*FGFR3*) or phosphatidylinositol-4,5-bisphosphate 3-kinase catalytic subunit alpha (*PIK3CA*) result in seborrheic keratosis (senile wart) [4] or epidermal nevi [162]. Recurrent somatic mutations in the PIK3-AKT signaling pathway that affect the cortical development result in hemimegalencephaly [163].

Lineage tracing studies have clearly illustrated the importance of the cell lineage of KC cancers. Although both cSCC and BCC are neoplasms of KC origin [21], their biological behaviors significantly differ from each other. BCC depends on the Sonic Hedgehog (SHH) pathway for its emergence [164,165], whereas cSCCs are dependent on EGFR-RAS signaling [158]. Forced overexpression of *KRas*^G12D^ in the interfollicular epidermis or hair follicle (HF) bulge stem cells produce papillomas, whereas that in the SHH-secreting HF matrix cells does not [166]. Discrete KC cancer lineages thus influence treatment efficacy for BCC, which makes a less amenable ICI target than cSCCs [167,168,169]. Therefore, as successful cross-talk between stem cells and the microenvironment (niche) determines the outcome of organoid structure development [170], the outcome of malignant progression [171] requires such gene–microenvironment interactions [139].

## 7. Immune Checkpoint Inhibition for cSCC

### 7.1. PD-1 Blockade For cSCC

The introduction of ICIs targeting the programmed death-1 (PD-1)/programmed death-ligand 1 (PD-L1) pathway or cytotoxic T-lymphocyte antigen 4 (CTLA-4) launched a revolution in anti-cancer therapy [16]. This strategy embodies the concept that the host’s exhausted pre-existing anti-tumor immunity can be reactivated. In particular, PD-1/PD-L1 blockade is considered to activate CTL-mediated tumoricidal responses at the effector phase and exhibit efficacy against myriad malignancies [172,173].

The first clinical report describing the efficacy of ICIs involved a case of metastatic cSCC in the lungs in an allogeneic renal transplant recipient [174]. Following the administration of the anti-PD-1 antibody (PD-1Ab) nivolumab, the patient experienced an 85% reduction in the tumor burden at the expense of steroid-refractory severe allograft rejection [174]. Subsequently, a case of locally advanced cSCC with nearly complete tumor regression after four cycles of treatment with the PD-1Ab pembrolizumab was reported [175]. This remarkable efficacy was reproducible in a case series in which five of six patients (83%) experienced a clinical response [176]. A phase 2 study of the PD-1Ab cemiplimab in a cohort of patients with locally advanced or metastatic cSCC revealed objective response (OR) rates of 44% [18] and 34.3–47% [19,177], respectively. A recently published case series has also proven similar findings, with OR rates of 34% [178]. Eastern cooperative oncology group performance status, rather than concurrent immunosuppression, affected the efficacy [178]. Overall, ICIs could offer promising therapeutic efficacy for advanced cSCC compared with the effects of conventional systemic therapy [179].

### 7.2. What Makes cSCC an Amenable Target for PD-1 Blockade?

UV exposure results in a high tumor mutational burden (TMB) and likely increases the susceptibility of cSCCs to ICI, as suggested by numerous studies of various malignancies [180,181,182,183,184,185]. Nevertheless, the efficacy of PD-1 blockade for cSCCs is unusually high compared to the OR rates of phase 2/3 studies of non-cutaneous SCCs, including cancers of the head and neck (13.3%) [186], esophagus (17–19%) [187,188], cervix (12.2%) [189] and lungs (20%) [190] (Table 6). This difference becomes more evident in primary studies in which ICI-treated cSCCs were analyzed in sub-clusters. The OR rate for cSCC lesions goes down from 50 to 56.7% to 24%, when the same regional sites have undergone two or more surgical procedures [18,191].

Clinical observations suggest that multiple cSCC recurrence episodes are associated with poorer clinical outcomes [13,192,193], including ICI resistance [18]. This presumably is closely associated with EMT [28] or immunoevasion [31,82]. Therefore, the presence of the so-called immunologically cold microenvironment [194,195] could compromise the host’s optimal tumoricidal activity, which needs to be reactivated in situ [16,196]. Contrarily, epidermal KCs, from which cSCC develop [166], could induce high immunogenicity.

#### 7.2.1. Immune–Anatomical Principle of the Squamous Epithelium

The skin epidermis is a stratified squamous epithelium facing an arid, harsh terrestrial environment. Terrestrial amniotes are armed with a specialized barrier, namely the stratum corneum (SC), to cope with such an essential requirement [197]. The major threat to the human epidermis is desiccation. We develop dry, scaly skin during the dry winter season, and skincare moisturizers alleviate such symptoms. Analogously, epidermal KCs produce lipids that prevent desiccation, and congenital defects in this machinery can sometimes manifest as severe, plate-like ichthyotic hyperkeratosis. The other important but less conspicuous feature compared to desiccation tolerance is structural integrity, which is maintained by the sulfur-rich proteinous deposition formed at the cell periphery, termed cornified cell envelopes (CEs) [198]. CEs stabilize the cytoskeleton and protect against myriad noxious and genotoxic stimuli, such as UVR [199]. Thus, akin to the mortar and the brick model, terminal differentiation of the epidermis, i.e., cornification, leads to a functional dichotomy in the SC [200]. Because defective epidermal differentiation is a hallmark feature of malignant progression [13] that reduces the response to PD-1 blockade [18], we started to suspect that the superior outcome of PD-1 blockade in patients with cSCCs may be attributable to the primary location of the tumor, namely the skin, which is covered by the SC [201]. Clinical observations could corroborate this notion, as the skin is often a target of GVHR, [7] drug toxicity [202] and irAEs [16,17,203], all of which are associated with extremely strong CTL-mediated immune responses.

#### 7.2.2. Contact Allergy and Topical Immunotherapy

Perhaps the epitome of such cutaneous CTL responses is contact hypersensitivity (CHS), which models the allergic contact dermatitis and utilizes both perforin/granzyme and Fas/Fas-ligand apoptotic pathways as effectors [204]. The mucosal tissue does not exhibit CHS but rather induces tolerance in a well-known immune–anatomical principle [205]. Given that the oral cavity, esophagus, vagina, rectum, anterior chamber of the eyes and epidermis are all covered by stratified squamous epithelium, it may not necessarily be illogical to infer that the unusually high immunogenicity of the dry-surfaced squamous epithelium could be attributable to the presence or absence of the SC [201]. An important fact is that the SC expresses the primary cytokine IL-1α [206]. This idea initially stemmed from clinical observations that the SC causes sterile inflammation [207], as observed in ruptured epidermal cysts or cystic acne [208]. Subsequently, it was demonstrated that SC extract exhibits high co-stimulatory activity and induces pyrexia/neutrophilia when intravenously injected into mice [209]. Therefore, epidermal differentiation (cornification) [200] appears to confer immunogenicity in the earliest afferent phase of local inflammatory responses [209], including CHS [210,211]. This may be an important explanation why topical therapy is feasible for cSCC (precursors) [12]. Alternatively, recent evidence regarding LC ontogeny may provide additional insight. Although LCs reside in squamous epithelia and exhibit similar transcriptomic signatures and functions [212], their ontogenic trajectories substantially differ depending on the niches (the epidermis or the squamous mucosa) [212] or the context (UV-damaged vs. steady-state epidermis) [213]. Therefore, it could be inferred that the epidermal differentiation program yields a fully cornified stratified squamous epithelium and renders superior immunogenicity through taking advantage of mononuclear phagocyte system’s plasticity [201,214].

## 8. Overcoming Immune Resistance

### 8.1. Microenvironmental Factors for Efficient Immune Checkpoint Blockade

It is inarguable that the pleiotropic cytokine TGF-β is one of the most important microenvironment-derived soluble factors in almost every aspect of cSCC pathology in that it initiates DNA damage responses [85], promotes EMT, [31,82,215], causes immunoevasion [31,216] and confers resistance to ICIs [18], as discussed previously.

The immunoedited [6,97] or immunologically cold microenvironment [194,195] could compromise the host’s optimal tumoricidal activity, which must be reactivated in situ [16,196]. Because successful PD-1 blockade requires a pre-existing immunologically hot microenvironment, cSCCs with multiple local recurrences are associated with poorer outcomes [18,191]. We conclude that the malignant behavior of tumor cells highly depends on the surrounding microenvironment or niche, which is the embodiment of the gene expression program [153,217,218,219]. Epigenomic changes could ultimately lead to the accumulation of ‘selfish’ genes [29,139], such as *HRAS*/*KRAS* [220] or *BRAF*^V600E^ [138], through altering gene expression program.

### 8.2. TGF-β Signalling Blockade

Despite their low TMB, cSCCs arise in sites of chronic inflammation, such as burn scars (Marjolin’s ulcer) or autosomal recessive dystrophic epidermolysis bullosa (RDEB) lesions, and these cancers are often invasive and metastatic [221]. In patients with RDEB, the absence of epidermal–dermal adhesion causes repeated episodes of scarring inflammation, which leads to epithelial migration/proliferation, fibrosis and extracellular matrix (ECM) remodeling while promoting the evolution of clones distinct from UV-associated cSCC [222]. In particular, the TGF-β signaling pathway plays a significant role in RDEB pathology by modulating ECM remodeling through cell–cell contact [223]. RDEB–cSCC cells are dependent on this intracellular signaling [224]. Despite the presence of EMT/immunoevasion in RDEB–cSCC cells [31,82], recent clinical observations suggest that PD-1 blockade holds promise [20,225]. However, this might not be the case if the microenvironment (niche) allows RDEB-cSCC cells to lose lineage commitment.

Recent clinical observations suggest that high mutational loads do not necessarily define the likelihood of response to PD-1 blockade in locally advanced/unresectable cSCCs [18,191] or metastatic melanomas [226,227]. This is in line with the fact that successful PD-1 blockade significantly alters gene expression programs [219,228,229] in the microenvironment (niche), in which immune responses arise [172,173]. Therefore, oncologists need to overcome the immunologically cold CTL-excluding microenvironment [228,229]. It is also known that suboptimal responses to PD-1 blockade are associated with TGF-β signaling signatures [100,228,230], supporting the legitimacy of manipulating the tissue factor [231,232]. Because most patients with cSCC die from poorly controlled local disease, rather than systemic metastatic spread [37], this approach will be of substantial benefit to such patients. Indeed, the angiotensin II type 1 receptor antagonist losartan counteracts the TGF-β signaling pathway, reduces ECM remodeling/fibrosis and ameliorates RDEB-associated cutaneous symptoms in mice [223]. However, systemic blockade of TGF-β could run the risk of severe autoimmune episodes given the phenotype of TGF-β–deficient mice [233].

TGF-β is stored as a pro–TGF-β precursor, and multiple post-translational modifications activates the TGF-b signaling [100]. Because immunosuppressive Tregs characteristically produce TGF-β upon T cell receptor stimulation [234], blockade of this immunosuppressive circuit [100] represents a legitimate approach to overcome the immune resistance program [219,228,229]. A promising approach is targeting a receptor for latent TGF-β, the biologically inactive form of TGF-β [100]. Antibodies raised against the membrane protein glycoprotein A repetitions predominant (GARP) inhibited intratumoral the Treg production of TGF-β and successfully eradicated PD-1 blockade-resistant tumors in mice [235]. A clinical trial of anti-GARP antibodies is currently underway [235].

## 9. Conclusions

Recent translational evidence revealed that dysregulated gene expression programs [152], rather than the mutational landscape per se [1,236], could define cancer tissue and immune responses [219,228,229]. Cell lineages/fates determine the development of a given structure [218] that subsequently tailors immune responses [231,232]. Analogously, cSCC, which arises from KCs upon lineage commitment for the fully cornified epidermis [166], is a more amenable target for PD-1 blockade than mucosal SCC or BCC [186,187,188,189]. By extension, the differentiation treatment of acute promyelocytic leukemia with tretinoin (all-*trans*-retinoic acid) [237] or acute myeloid leukemia with inhibitors of FMS-like tyrosine kinase 3 [238] reprograms gene expression. However, this sometimes provokes the differentiation syndrome. Excessive blood neutrophil production predicts a poorer clinical outcome both in differentiation therapies [239] and PD-1 blockade [240]. Therefore, targeted therapeutic measures in the next generation need to effectively divert the cancer gene expression program, as has been proved in PD-1 blockade [219,228,229].

DNA/histone modifications, which are located distally to the genome sequences, highly influence gene expression programs. Since the discovery of DNA methylation in 1980 [241], the epigenetics has been the subject of intensive investigations and potentially makes an attractive potential therapeutic target [242]. Extensive global reprogramming of epigenetic patterns, such as gain/loss in DNA methylation or changes to histone marks (acetylation/phosphorylation), characterize malignancies [243]. At the DNA level, hypermethylation of GC-rich promoter sequences can downregulate tumor suppressor genes. At the histone level, hyperacetylation/hypomethylation loosens the chromatin structure, leading to the chromosomal instability. Although hypermethylation in cancer genomes alter histone modifications and thus gene expression programs, the efficacy of DNA methyltransferase inhibitors was not as striking as expected in myeloproliferative disorders [242]. Whether this is also the case with solid tumors remains unclear to date [244]. Epigenetic modification of T cell, as well as tumor cell could another attractive way to overcome the immune resistance [245]. It is already known that *PD1* gene promoter demethylation is imprinted during the effector phase of CTL exhaustion in mice [246], and the chromatin accessibility of circulating CD8^+^ T cells [247] or CTLA4 methylation [248] determines the outcome of ICI in humans. Preclinical studies have shown that low-dose administration of the demethylating agent decitabine rejuvenates the cytotoxic activity and overcomes immunosuppression associated with chronic viral infection [249]. Although these pieces of evidence hold promise for epigenetic therapies in combination with ICIs, the key for successful intervention would depend on the timing and the circumstances [218]. The successful clinical application of this legitimate, mechanism-based disease control strategy may await additional measures, such as efficient and reproducible biomonitoring or accurate drug delivery.

By reviewing the current knowledge about cSCC from multiple perspectives, we realize that cellular immune responses are the key to effective cancer immunosurveillance. As HIES denotes, AD is characterized by broad defects in the epidermal differentiation program (cornification) [250], which potentially fails to imprint the innate immunological memory (CHS) [155,201,210,214,251]. Although this hypothesis is largely based on inference at the moment [201,214], future translational studies based on the important lessons from the bedside may uncover the exact mechanism involving the epimmunome [252] or epidermal immune microenvironment [253].

## Figures and Tables

**Table 1:** Summary of the BWH and AJCC eighth edition classification systems for cutaneous squamous cell carcinoma.

Summary of the BWH and AJCC8 Tumour Classification System	
AJCC 8th Edition	
T1	<2 cm in greatest diameter
T2	≥2 cm but <4 cm in greatest diameter or minor bone invasion or perineural invasion or deep invasion ^a^
T3	≥4 cm in greatest diameter or minor bone invasion or perineural invasion or deep invasion ^a^
T4a	Tumour with gross cortical bone and/or marrow invasion
T4b	Tumour with skull bone invasion and/or skull base foramen involvement
BWH	
T1	0 High-risk factors ^b^
T2a	1 High-risk factor
T2b	2–3 High-risk factors
T3	4 High-risk factors or bone invasion

Abbreviations: AJCC, American Joint Committee on Cancer; BWH, Brigham and Women’s Hospital; T, tumour stage from TNM staging system. ^a^ Deep invasion defined as invasion beyond the subcutaneous fat or >6 mm (as measured from the granular layer of adjacent normal epidermis to the base of the tumour), perineural invasion defined as tumour cells in the nerve sheath of a nerve lying deeper than the dermis or measuring 0.1 mm or larger in calibre or presenting with clinical or radiographic involvement of the aforementioned nerves without skull base invasion or transgression. ^b^ BWH high-risk factors include tumour diameter ≥ 2 cm, poorly differentiated histology, perineural invasion of nerve(s) ≥0.1 mm in calibre or tumour invasion beyond subcutaneous fat (excluding bone invasion, which upgrades the tumour to BWH stage T3).

**Table 2:** Genetic predisposing factors of cutaneous squamous cell carcinoma. Genetically defined conditions are listed.

Genetic Predisposing Factors for cSCC				
Condition	Chromosome	Gene	Function	OMIM
**Defective DNA Repair**				
Xeroderma pigmentosum (XP)				
XPA	9q22	*XPA*	DNA repair	278700
XPB	2q14	*ERCC3*	DNA repair	610651
XPC	3p25	XPC	DNA repair	278720
XPD	19q13	*ERCC2*	DNA repair	278730
XPE	11p11	*DDB2*	DNA repair	278740
XPF	16p13	*ERCC4*	DNA repair	278760
XPG	13q33	*ERCC5*	DNA repair	278780
XP variant (XPV)	N/A	*POLH*	DNA repair	278750
Fanconi anaemia (FA)	…	…	Interstrand cross-link repair	…
Dyskeratosis congenita	…	…	Telomere maintenance	…
Muir–Torre syndrome	2p21-p16/3p22.2	*MSH2*/*MLH1*	DNA mismatch repair	158320
**Primary immunodeficiency**				
Epidermodysplasia verrciformis	…	…	…	…
GATA2 deficiency/MonoMAC syndrome	3q21.3	*GATA2*	Monocyte/B-cell/NK cell maintenance	614172
WHIM syndrome	2q22.1	CXCR4	BM release of PMNs	193670
Hyper-IgE recurrent infection syndrome (HIES)				
HIES1	17q21.2	*STAT3*	Th17 differentiation	147060
HIES2	1q21.3	*DOCK8*	Th17 differentiation	611432
HIES3	20q11.22	*ZNF341*	Th17 differentiation	618282
HIES4	5q11.2	*IL6ST*	Th17 differentiation	618523
HIES5	1q21.3	*IL6R*	Th17 differentiation	618944
**Impaired TGF-b signalling pathway**				
Self-healing multiple squamous epithelioma (Ferguson–Smith disease)	9q22.33	*TGFBR1*	Autocrine/paracrine maintenance of TGF-b signalling	132800

https://www.omim.org (Last access date: 30 January 2021). Abbreviations: MonoMAC, monocytopenia and mycobacterial infection; NK, natural killer, BM, bone marrow; PMN, polymorphonuclear neutrophil. Gene symbols: Please refer to the outer source.

**Table 3:** Recurrently mutated genes in cSCCs.

Recurrently Mutated Genes in cSCC		
Gene	Function	Reference
*TP53*	Tumour suppressor	[3,115,116,117]
*NOTCH1*	Regulation of multiple differentiation processes	[3,115,116,117]
*NOTCH2*	Regulation of multiple differentiation processes	[3,115,116,117]
*CDKN2A*	G1/S checkpoint	[115,116,117]
*HRAS*	GTPase	[115,116,117]
*NF1*	RasGAP	[3,116]
*PTEN*	Tumour suppressor	[116,117]
Gene symbols: Please refer to the outer source.		

**Table 4:** Genetic predisposition factors for cutaneous melanoma. Genetically defined conditions are listed.

Genetic Predisposing Factors for Cutaneous Melanoma		
Gene	Function	Remarks
*CDKN2A*	G1/S checkpoint	Melanoma and neural system tumour syndromeMelanoma–pancreatic cancer syndrome
*CDK4*	Cell cycle progression (G1-S/G2-M)	
*MC1R*	Pigment regulation	
*XRCC3*	DNA repair	
*MITF*	Transcription factor	
*TERT*	Telomere maintenance	
*POT1*	Telomere maintenance	

https://www.omim.org (Last access date: 30 January 2021). Gene symbols: Please refer to the outer source.

**Table 5:** The RASopathies. Note that individual germline mutations in the mitogen-activated protein kinase (MAPK) pathway can cause distinctive disease manifestations.

The RASopathies					
Syndrome	Chromosome	Gene	Function	Skin Pigmentation	Cancer Predisposition
Cardio-facio-cutaneous syndrome	7q34	*BRAF*	Kinase	Yes	Unclear
	15q22.31	*MAPK1*	Kinase		
	19p13.3	*MAPK2*	Kinase		
	12p12.1	*KRAS*	GTPase		
Neurofibromatosis Type 1	17q11.2	NF1	RasGAP	Yes	Yes
Noonan Syndrome	12q24.1	*PTPN11*	Phosphatase	No	Yes
	2p22.1	SOS1	RasGEF		
	3p25.1	*RAF1*	Kinase		
	12p12.1	*KRAS*	GTPase		
	1p13.2	*NRAS*	GTPase		
	10q25.2	*SHOC2*	Scaffolding		
	11q23.3	*CBL*	E3 ubiquitin ligase		
Noonan syndrome with multiple lentigines	12q24.1	PTPN11	Phosphatase	Yes	Unclear
	3p25.1	*RAF1*	Kinase		
Capillary malformation-arteriovenous malformation	5q14.3	*RASA1*	RasGAP	No	Yes
Costello syndrome	11p15.5	*HRAS*	GTPase	No	Yes
Legius syndrome	15q14	*SPRED1*	SPROUTY-related, EVH1 domain-containing protein 1	Yes	No

Gene symbols: Please refer to the outer source.

**Table 6:** The outcomes of clinical trials of programmed death-1 (PD-1) blockade for squamous cell carcinomas (SCCs).

Outcomes of Clinical Trials of PD-1 Blockade for SCCs						
Primary lesion	Condition	Drug	Phase	OR (%)	ClinicalTrials. gov# (NCT#)	Reference
Cutaneous	Locally advanced/metastatic	Cemiplimab	1	50 (30–70)	02383212	[197]
Cutaneous	Metastatic	Cemiplimab	2	47 (34–61)	02760498	[197]
Cutaneous	Locally advanced	Cemiplimab	2	44 (32–55)	02760498	[18]
Cutaneous	Recurrent/metastatic	Pembrolizumab	2	34.3 (25.3–44.2)	03833167	[19]
Head and neck	Recurrent^*1^	Nivolumab	3	13.3 (9.3–18.3)	02105636	[206]
Oesophagus	Advanced, treatment-refractory^*2^	Nivolumab	2	17 (10–28)	02569242	[207]
Oesophagus	Advanced, treatment-refractory^*3^	Nivolumab	3	19 (14–26)	03143153	[208]
Cervix	Advanced, treatment-refractory^*4^	Pembrolizumab	2	14.6 (7.8–24.2)	02628067	[209]
Lung	Advanced, treatment-refractory^*5^	Nivolumab	3	20 (14–28)	01642004	[210]

*1: Disease progression within 6 months after platinum-based chemotherapy. *2: SCC, adenosquamous cell carcinoma (Ad-SCC), or adenocarcinoma (unresected or resected) that was refractory or intolerant to fluoropyrimidine-based, platinum-based and taxane-based chemotherapy. *3: Unresectable advanced/recurrent SCC/Ad-SCC refractory/intolerant to one previous fluoropyrimidine-based and platinum-based chemotherapy. *4: Previously treated with chemotherapy, recurrent/metastatic disease. *5: Head-to-head trial with docetaxel. Stage IIB/IV non-small cell lung cancers with recurrence after at least one prior platinum-based regimen.

## Abbreviations

AD	atopic dermatitis
AJCC	American Joint Committee on Cancer
AJCC8	8th Edition of AJCC staging system
AK	actinic keratosis
BCC	Basal cell carcinoma
BM	bone marrow
BWH	Brigham and Women’s Hospital
CDKN2A	cyclin-dependent kinase inhibitor 2A
CE	cornified cell envelope
CFC	Cardio-Facio-Cutaneous
CHS	contact hypersensitivity
CIB1	calcium and integrin binding 1
cSCC	cutaneous squamous cell carcinoma
CTL	cytotoxic T lymphocytes
CTLA-4	cytotoxic T-lymphocyte antigen 4
CXCR	C-X-C chemokine receptor
DN	dysplastic nevus
ECM	extracellular matrix
EGFR	epidermal growth factor receptor
EMT	epithelial-mesenchymal transition
EV	epidermodysplasia verruciformis
FA	Fanconi anaemia
FLT3	FMS-like tyrosine kinase 3
FSD	Ferguson-Smith disease
GARP	glycoprotein A repetitions predominant
GATA2	GATA binding protein 2
GOF	gain-of-function
GTP	guanosine triphosphate
GVHR	graft-versus host reaction
HER2	human epidermal growth factor receptor 2
HIES	hyper-IgE recurrent infection syndrome
HIV	human immunodeficiency virus
HPV	human papilloma virus
ICI	immune checkpoint inhibitor
interleukin	IL
irAE	immune-related adverse event
KA	keratoacanthoma
KC	keratinocyte
LC	Langerhans cell
LOF	Loss-of-function
MAPK	mitogen-activated protein kinase
MEK1	MAP2K1
MEK2	MAP2K2
MN	melanocytic nevus
MonoMAC	monocytopenia and mycobacterial infection
NOTCH1	notch receptor 1
OCA	oculocutaneous albinism
OR	objective responses
PD-1	programmed death-1
PD-1Ab	anti-PD-1 antibody
PD-L1	programmed death-ligand 1
PID	primary immunodeficiency
PMN	polymorphonuclear neutrophil
PTEN	phosphatase and tensin homolog
PY	people/year
R	receptor
RDEB	recessive dystrophic epidermolysis bullosa
RTKI	receptor tyrosine kinase inhibitors
SC	stratum corneum
SCID	severe combined immune deficiency
SEER	Surveillance, Epidemiology, and End Results
SHH	Sonic Hedgehog
SOX10	sex determining region Y-box 10
ST	signal transducer
STAT	signal transducer and activator of transcription
TERT	telomerase reverse transcriptase
TGF-b	transforming growth factor beta
Th	T helper
TMB	tumour mutational burden
TP53	tumour protein 53
TYK2	tyrosine kinase 2
UICC	Union for International Cancer Control
UV	ultraviolet
UVR	UV radiation
WHIM	Warts, Hypogammaglobulinemia, Infections, Myelocathexis
